# Supplementary material for: Effectiveness of online practical education on vaccination training in the students of bachelor programs during the Covid-19 pandemic
Source: PLoS One. 2023 Jan 12;18(1):e0280312. doi: 10.1371/journal.pone.0280312 (PMC9836285; doi:10.1371/journal.pone.0280312)
Supplement: Attachment — Submitted filename: Response to Reviewers.docx [file pone.0280312.s003.docx]

Author’s response to reviews

# Title: Effectiveness of online practical education on vaccination training in the students of bachelor programs during the Covid-19 pandemic

**Date:** 18 August 2022

We thank all the Reviewers for their valuable feedback and taking the time to provide useful comments to improve our manuscript entitled “Effectiveness of Online Practical Education on Vaccination Learning in the apprenticeship stage of Bachelor students in the Covid-19 Pandemic”. Based on the constructive comments the following changes have been made:

It is necessary to explain that the corrections considered by honorable reviewers specified with the yellow highlight in the text of the manuscript.

***Journal Requirements:***

1- Please ensure that your manuscript meets PLOS ONE's style requirements, including those for file naming.

**Response:** Thanks for the valuable comments, the requested corrections were made

2- Please provide additional details regarding participant consent.

**Response:** The additional details regarding participant consent were added in Method section, page5, line 101-108

3- We suggest you thoroughly copyedit your manuscript for language usage, spelling, and grammar.

**Response:** Thanks for the valuable comments of the esteemed Editor that helped to improve the details of the study. The language of manuscript was revised. The track change file of language editing Institute attached as “supporting information” file.

4- In your Data Availability statement, you have not specified where the minimal data set underlying the results described in your manuscript can be found.

**Response:** Minimal data set of present study uploaded as “Supporting Information” files

5. We note that the grant information you provided in the ‘Funding Information’ and ‘Financial Disclosure’ sections do not match.

**Response:** The grant information statement was removed from the manuscript and modified in the submission system. Due to the fluctuation of the price of the dollar against the Rial (Iranian currency), the amount that was previously registered in the system was modified according to the current price of the dollar in Iran.

***Response to Reviewer 1: Dr Syeedun Nisa***

1-Lot of Grammatical problems is there and whole text needs editing.

**Response:** Thanks for the valuable comments of the reviewers that helped to improve the details of the study. The language of manuscript was revised. The track change file of language editing Institute attached.

2-Apart from T-test, some advanced statistical tests can be applied to make it more technically sound.

**Response:** Thank you for your valuable comment. We examined all variables based on literature that may have an impact on students' knowledge, attitude, self-efficacy and performance.

The results showed that there is no statistically significant difference between the intervention and control groups in terms of these variables before the study.

Therefore, it can be concluded that the difference in the mean scores of knowledge, attitude, self-efficacy and performance between the intervention and control groups is due to the intervention.

We added this sentence in the limitations.

It is possible that there have been variables that have not been measured in this study. This issue is one of the characteristics of cohort intervention studies.

Also, we reviewed many experimental studies (1-10), and independent t-test was used in all of these studies. A sample of studies is given below.

1.	Theodosi S, Nicolaidou I. Affecting young children’s knowledge, attitudes, and behaviors for ultraviolet radiation protection through the internet of things: a quasi-experimental study. Computers. 2021;10(11):137.

2.	Öz GÖ, Ordu Y. The effects of web based education and Kahoot usage in evaluation of the knowledge and skills regarding intramuscular injection among nursing students. Nurse Education Today. 2021;103:104910.

3.	Craig SJ, Kastello JC, Cieslowski BJ, Rovnyak V. Simulation strategies to increase nursing student clinical competence in safe medication administration practices: A quasi-experimental study. Nurse Education Today. 2021;96:104605.

4.	Grønlien HK, Christoffersen TE, Ringstad Ø, Andreassen M, Lugo RG. A blended learning teaching strategy strengthens the nursing students’ performance and self-reported learning outcome achievement in an anatomy, physiology and biochemistry course–A quasi-experimental study. Nurse Education in Practice. 2021;52:103046.

5.	Chang H-Y, Wu H-F, Chang Y-C, Tseng Y-S, Wang Y-C. The effects of a virtual simulation-based, mobile technology application on nursing students’ learning achievement and cognitive load: Randomized controlled trial. International Journal of Nursing Studies. 2021;120:103948.

6.	Putra A, Sumarmi S, Sahrina A, Fajrilia A, Islam M, Yembuu B. Effect of Mobile-Augmented Reality (MAR) in digital encyclopedia on the complex problem solving and attitudes of undergraduate student. International Journal of Emerging Technologies in Learning (IJET). 2021;16(7):119-34.

7.	Ma X, Yang Y, Chow KM, Zang Y. Chinese adolescents’ sexual and reproductive health education: A quasi‐experimental study. Public Health Nursing. 2022;39(1):116-25.

8.	Sarker R, Islam M, Moonajilin M, Rahman M, Gesesew HA, Ward PR. Effectiveness of educational intervention on breast cancer knowledge and breast self-examination among female university students in Bangladesh: a pre-post quasi-experimental study. BMC cancer. 2022;22(1):1-7.

9.	Kandula UR, Philip D, Mathew S, Subin A, Godphy A, Alex N, et al. Efficacy of video educational program on interception of urinary tract infection and neurological stress among teenage girls: An uncontrolled experimental study. Neuroscience Informatics. 2022;2(3):100026.

10.	Permatasari TAE, Rizqiya F, Kusumaningati W, Suryaalamsah II, Hermiwahyoeni Z. The effect of nutrition and reproductive health education of pregnant women in Indonesia using quasi experimental study. BMC Pregnancy and Childbirth. 2021;21(1):1-15.

If the esteemed reviewer has a specific statistical analysis in mind say its name. We will do.

3. Authors are advised to support data with existing literature.

Response: The results of this study were compared with similar studies in the discussion section.

***Response to Reviewer 2:***

1-This is an interesting study and the author have collected a unique dataset using cutting edge methodology. However, I recommend that the conclusion and discussion, as well as the recommendation based on the observations, could be more detailed.

Response: Thanks for the valuable comments of the esteemed reviewer that helped to improve the study; more details were provided in section of conclusion and discussion, as well as the recommendation based on the observations.
