# Supplementary material for: Effectiveness of online practical education on vaccination training in the students of bachelor programs during the Covid-19 pandemic
Source: PLoS One. 2023 Jan 12;18(1):e0280312. doi: 10.1371/journal.pone.0280312 (PMC9836285; doi:10.1371/journal.pone.0280312)
Supplement: Attachment — Submitted filename: Response to Reviewers.docx [file pone.0280312.s004.docx]

Author’s response to reviews

**Title:** Effectiveness of online practical education on vaccination training in the students of bachelor programs during the Covid-19 pandemic

**Authors:**

Samane Shirahmadi,

Seyed Mohamad Mehdi Hazavehei

Hamid Abbasi,

Marzie Otogara,

Tahere Etesamifard,

Ghodratolah Roshanaei,

Neda Dadaei

Malihe Taheri*

**Version:** 2

**Date:** 2022 Des 10

**Author's response to reviews:** see over

We thank all the Reviewers for their valuable feedback and taking the time to provide useful comments to improve our manuscript entitled “Effectiveness of online practical education on vaccination training in the students of bachelor programs during the Covid-19 pandemic.” Based on the constructive comments the following changes have been made.

***Response to Reviewer 3:***

1. It is better that in abstract result mentioned numerical result .for example prevalence of group1 vs group 2 with p value=?

Response: Thank you for your valuable comment. We have revised the abstract result section.

2. The methods need to be improved by providing more detail information related to participant’s selection (e.g. respond rate; necessary permissions from who? How did the researcher contact the potential participants?)

Response: Thank you for your valuable comment. We have revised the method section. Page…, line…..

3. Discuss more about your sampling strategy? The structure of your sampling is so vague and understandable. Did you have sampling frame? How did you access to this frame

Response: Thank you for your valuable comment. We have revised the method section and sampling strategy.

We have added these paragraphs in in method section:"

Page7.

4. It was better if you could show the process of samples selection and methods using a flowchart with consort format.

Response: Thank you for your valuable comment. We have added flowchart of sampling with consort format (Fig 1).

5. What are the data extract’s center characteristics? Is it governmental or private, is it referral or not referral and so on, discuss more about it

Response: Thank you for your valuable comment. All the data were extracted from public health undergraduate students of Hamadan University of Medical Sciences. Hamadan University of Medical Sciences is a public university.

6. How many observers did you have? If you had more than one observer, you must mention agreement index like kappa coefficient (write in method section)

Response: Thank you for your valuable comment. There was only one observer. We have added in the method section. Page 8, line 183

7. Write about all applied exclusion and inclusion criteria a bit more clearly by which you selected samples for this survey.

Response: Thank you for your valuable comment. We have Added exclusion and inclusion criteria in the method section. Page 7-8.

**Response to Reviewer 4:**

1. The control group was selected from students in the previous year because there was no one passing the in-person training this year. But students can accumulate practical experience or even forget about the knowledge in the class after one year, which may affect the results of knowledge, attitude, and self-efficacy. Is this the best choice for the control group to reflect the video training effectiveness?

Response: Thank you for your valuable comment. We have completed the selection of the control group. Page 8, lines 175-178.

2. Some grammar errors in the main text need to be corrected, e.g. line 86-88, line 247. Odd and inconsistent capital letters e.g. in Table 2 columns, line 91, 243, 257, 266 and so on, need to be corrected, too. Please carefully proofread the manuscript.

Response: Thank you for your valuable comment. We have edited errors.
